# Towards enhanced *Sargassum* monitoring in the Caribbean Sea

**DOI:** 10.1038/s41598-025-93001-9

**Published:** 2025-03-15

**Authors:** Javier Arellano-Verdejo, Hugo E. Lazcano-Hernandez, Jorge Prado Molina, Uriel de Jesús Mendoza Castillo, Víctor Manuel Jiménez Escudero, Francisco Javier Osorno Covarrubias, Gabriela Gómez Rodríguez, José Antonio Quintero Pérez, Steven Czitrom Baus, Iván Penié Rodríguez, Guadalupe Tapia Varela, Ilma Maclovia Huy Domínguez

**Affiliations:** 1 Department of Observation and Study of the Earth, Atmosphere, and Ocean, El Colegio de la Frontera Sur, 77014 Chetumal, Mexico; 2 Department of Observation and Study of the Earth, Atmosphere, and Ocean, CONAHCYT-ECOSUR, 77014 Chetumal, Mexico; 3 Instituto de Geografía UNAM, National Laboratory for Earth Observation, 04510 Mexico City, Mexico; 4 Academic Unit of Territorial Studies Yucatan, Instituto de Geografía UNAM, 97000 Merida, Mexico; 5 Oceanic and Coastal Processes Academic Unit, Instituto de Ciencias del Mar y Limnología UNAM, 04510 Mexico City, Mexico; 6 Research Coordination, Oceanus International, Zilk 64, 2191 AM De Zilk, The Netherlands

**Keywords:** *Sargassum* monitoring, Platform monitoring, Early warning system, Caribbean Sea, Algal bloom, Information technology, Software, Computational platforms and environments, Data integration, Data processing, Image processing, Software

## Abstract

Monitoring *Sargassum* along the coasts of the Greater Caribbean has become essential due to recurrent blooms since 2018, which bring severe ecological, economic, and social impacts that accumulate yearly. Developing an advanced, monitoring platform would enable affected countries to make informed decisions, manage critical zones, and mitigate negative impacts on coastal ecosystems, economies, and public health. In this work, we present the LANOT platform, a new tool for monitoring *Sargassum* across the Mexican Caribbean and neighboring regions, including Belize, Guatemala, and parts of Honduras. Utilizing Sentinel-2 and Landsat-8/9 satellite imagery, the platform provides data updated every five days and includes interactive features for selecting layers, querying *Sargassum* imagery by date or raft area, measuring regions, and downloading files in GeoTIFF, GeoJSON, and PNG formats. These interactive tools allow users to create and download custom files for use in their *Sargassum* management plans. Although the platform faces inherent limitations of satellite remote sensing, it represents a key advancement in monitoring efforts, marking a milestone for *Sargassum* observation in the region and supporting regional ecosystem management and research.

## Introduction

Although they occur in the open ocean, algal blooms can have significant ecological and anthropogenic impacts on coastal areas. This has been the case with pelagic *Sargassum* blooms in the equatorial Atlantic Ocean, which ultimately wash ashore along the coasts and beaches of the Caribbean Sea. These beaching events have been observed since 2011, and have led to the identification of the great Atlantic *Sargassum* belt (GASB)^1^, which extends from West Africa to the Gulf of Mexico (across the Intra-Americas Sea and the tropical Atlantic Ocean). The GASB has contributed to the large numbers of *Sargassum* stranding on Caribbean beaches, which in 2018 reached the highest recorded influx of pelagic *Sargassum* in the region^1^.

Pelagic *Sargassum* refers to a type of macroalgae that includes two species, *Sargassum* natans and *Sargassum* fluitans. These algae freely float on the ocean surface throughout their entire life cycle^2,3^, and the scientific community collectively refers to these two species as *Sargassum* spp. *Sargassum* typically distributes itself in the Sargasso Sea, the northern Caribbean Sea, and the Gulf of Mexico, forming a “*Sargassum* migratory loop” that follows a clockwise circulation pattern^4^. In the open ocean, *Sargassum* forms dense aggregations known as “rafts,” which represent a unique floating ecosystem where surface nutrients and substrates are scarce^2^. These *Sargassum* rafts support a diverse array of invertebrates, fish, and turtles^5,6^, providing critical habitats for shelter, feeding, and breeding for numerous species of ecological and commercial importance^7^.

Focusing on the observation of *Sargassum* from space, the challenges associated with this activity are those intrinsic to satellite remote sensing. These challenges include the physical phenomena experienced by electromagnetic waves as they travel through Earth’s atmosphere, as well as the technological limitations of current satellite platforms and sensors, which affect the availability and quality of satellite images. For example, the orbits of the satellite platforms determine the spatial and temporal resolutions of the images, while the spectral and radiometric characteristics are linked to the sensors on board the satellites^8^. It is therefore crucial to develop methodologies that can extract valuable information from the vast archive of satellite images. This is the area in which post-processing, analysis, and visualization efforts make significant contributions.

On the other hand, the ecological and socio-economic challenges posed by *Sargassum* arise when the macroalgae become stranded on beaches. To carry out these tasks with increasing precision, there is a requirement for accurate and simplified information, such as that offered by early warning systems. Given the enormous quantity of *Sargassum* stranding along hundreds of kilometers of coastline, its removal cannot be achieved in sufficient quantity or with the necessary speed. It therefore has the potential to cause negative impacts on coastal ecosystems and human activities^9^. For example, *Sargassum* decomposition can pose a threat to human health since it produces high concentrations of hydrogen sulfide ($H_2S$) and ammonia ($NH_3$). Under prolonged exposure, ammonia can lead to upper respiratory tract irritation, headaches, nausea, and, in extreme cases, pulmonary, neurological, and cardiovascular damage due to hypoxia^10^. Furthermore, in common with other algae, *Sargassum* absorbs dissolved elements from the ocean water, such as metals and metalloids^11^, which necessitates further studies to determine its safe use and final disposal. This is critical because, if *Sargassum* is to be used for food or pharmaceutical products, the concentration of these elements could pose a health risk. Moreover, the leachates generated during the decomposition of macroalgae can also contaminate the soil where it is accumulated^9^. However, due to the significant ecological and social impacts of these massive *Sargassum* influxes, there is now an urgent need for higher spatial resolution monitoring (at a pixel size of approximately 1 km or smaller) near these coastal areas. Such detailed observations could help support the development of an early warning system, which would, in turn, improve the management, utilization, and final disposal of *Sargassum*.

Finally, as with any other type of biomass, *Sargassum* has an energetic value and presents attractive opportunities for conversion into biogas through anaerobic digestion. Primarily composed of methane, biogas has a high energy value and can be utilized to meet various energy needs^12^. In addition, according to^13–15^ clean and dry *Sargassum* has the potential to be used in the production of fertilizer, compost, sodium alginate for food and pharmaceutical products, construction blocks for housing, paper production, beauty products, agricultural, livestock products, bioplastics, materials for the absorption of dissolved contaminants in water, and biogas^16,17^.

However, this requirement represents one of the major practical challenges. To harvest *Sargassum* in open waters, it must first be located, and specialized boats, equipment, and personnel are required to perform the task. These considerations highlight the importance of *Sargassum* monitoring for understanding marine life, studying coastal ecosystems, and improving the quality of life of the people directly affected by the impact of this phenomenon. However, given the vast areas in which *Sargassum* can be found in open waters and the large quantities that strand along hundreds of kilometers of coastline, its observation, monitoring, quantification, management, use, and final disposal pose challenges that require the involvement of multidisciplinary teams.

## *Sargassum* monitoring platforms

A *Sargassum* monitoring platform is an integrated system of technologies and methods designed to detect, track, and assess the presence and accumulation of *Sargassum* in marine and coastal areas. The objective of these platforms is to provide accurate, near real-time data to facilitate decision-making for authorities, scientists, environmental managers, and local communities, with the aim of mitigating the adverse effects of *Sargassum* on ecosystems, the economy, and society. However, due to the dynamic behavior and widespread distribution of *Sargassum* rafts in the ocean, and the physical and technological limitations of observing them at different scales, it is necessary to develop monitoring platforms that can address various aspects of these challenges.

In terms of utility, we consider that a *Sargassum* monitoring platform should have the capacity to provide coverage at varying geographical scales. This implies monitoring vast areas in open waters (oceanic level) while simultaneously focusing on specific sectors along the coast and beaches, adapting to the specific management requirements of each context. Integrating diverse technologies is crucial in this regard, combining satellite observation systems that allow broad, continuous large-scale surveillance with more detailed tools such as drones and in-situ sensors that can provide high-resolution data in critical or specific areas of interest.

A *Sargassum* monitoring platform must also ensure the collection of data with minimal latency, enabling rapid responses to be made to critical events. The data obtained must be accurate and reliable, which requires constant calibration and validation of detection systems, as well as the use of image and data processing algorithms. The platform must also be flexible and adaptable to different environmental and operational conditions to ensure its effectiveness in a dynamic and complex environment, such as the Mexican Caribbean.

Finally, the platform should promote collaboration and information exchange among multiple stakeholders, facilitating the integration of data from various sources, including data generated through citizen science. This will help create a robust, inclusive, and effective monitoring system that allows a more coordinated and efficient response to the challenges posed by *Sargassum* accumulation.

Although no agreement exists on how to classify *Sargassum* monitoring platforms at the time of writing this article, we believe that at least two factors should be considered for this purpose:

- Source of data used for monitoring
- Area of coverage

### *Sargassum* monitoring platforms based on data type

Depending on the data source, *Sargassum* monitoring platforms may utilize the following: medium- and high-resolution satellite imagery, very high-resolution imagery acquired by drones, data collected from buoys and in situ sensors, and, finally, data gathered through citizen science initiatives. Each of these data sources offers distinct advantages and limitations, as outlined in Table 1.

#### Satellite image-based monitoring platforms

Satellite image-based platforms, such as those provided by NASA (Terra, Aqua, and Landsat) or ESA (Sentinel), among other space agencies, have become indispensable tools for monitoring *Sargassum*. These platforms provide moderate-resolution (Terra, Aqua) and high-resolution (Landsat and Sentinel) images covering vast geographic areas, enabling the detection of extensive accumulations of *Sargassum* in open waters. The main advantage of satellite imagery is its capacity to provide comprehensive and consistent coverage, facilitating the observation of extensive ocean areas. These platforms also offer access to historical data, which permits long-term trend analysis and the effective planning of preventive measures. However, it should be noted that they also have certain limitations. The spatial resolution of the images may be inadequate for the detection of minor *Sargassum* accumulations near the coast. Moreover, meteorological conditions, such as cloud cover, can impact the quality and availability of the images. A further challenge is the difficulty of performing real-time monitoring given the time required for data acquisition and processing.

Furthermore, satellite image-based platforms used for the observation and monitoring of *Sargassum* rely mainly on ocean color indices to identify the presence of *Sargassum* in the images. The most commonly-used indices include the Maximum Chlorophyll Index (MCI)^18^, Floating Algae Index (FAI)^19^, and Alternative Floating Algae Index (AFAI)^20^.

#### Drone image-based monitoring platforms

The use of unmanned aerial vehicles, or drones, represents another innovative strategy for *Sargassum* monitoring. This technology enables the capture of very high-resolution images at low altitudes, thereby facilitating the detection of *Sargassum* in detail in specific coastal areas^21,22^. The main advantage of the use of drones is their flexibility and capacity for targeted monitoring in inaccessible locations. They can also provide near real-time data, which is vital for rapid decision-making in *Sargassum* management. Nevertheless, the use of drones does have limitations. Their coverage is limited compared to that of satellite platforms since they can only monitor relatively small areas in a single flight. Drones also require trained operators and the acquisition of specific permits, which can act to increase operational costs. Moreover, their flight autonomy is constrained by battery life, which restricts the extent of the areas that can be monitored in a single mission. They are also vulnerable to extreme weather conditions.

An example of beach images acquired by drones is provided by the academic service for meteorological and oceanographic monitoring (SAMMO) of the Institute of Marine Sciences and Limnology of UNAM^23^. Although this website is not an early warning platform, it offers a collection of aerial images of beaches in the municipality of Puerto Morelos, Quintana Roo, Mexico, which have been useful for studies aimed at quantifying *Sargassum*^22^.

#### In situ sensor-based monitoring platforms

In situ monitoring platforms, including oceanographic buoys, drifters, gliders, and expendable bathythermographs (XBT), are used to continuously collect real-time data on water quality, temperature, salinity, chlorophyll concentration, and other environmental factors that can further our understanding of *Sargassum* proliferation and, in some cases, facilitate its observation. Despite this, in situ sensor-based platforms also present limitations. Their coverage is limited since they can only provide data from the specific locations where the instruments are situated. Furthermore, the installation and maintenance of these platforms can be costly due to the challenging maritime conditions in which they are often deployed, and they are susceptible to damage from adverse weather events or even vandalism. The NOAA Coast Watch and AOML Ocean Observations Viewer platforms complement the information provided by USF and the MCI sensor, with data from various in situ sensor platforms. Furthermore, websites such as SIMAR^24^ and CARICOOS^25^ present data layers collected by virtual buoys on their maps.

#### Citizen science-based monitoring platforms

Citizen science plays a significant role in *Sargassum* monitoring. Platforms that engage volunteers, such as those incorporating mobile apps and community networks^26^, enable the collection of in situ data related to the presence and movement of *Sargassum*^27^. These platforms are invaluable for covering large areas at relatively low costs, while also fostering community participation in addressing the issue. However, citizen science initiatives do face challenges in terms of the quality and accuracy of the data collected, as they rely on the training and commitment of volunteers. Furthermore, the lack of standardization in data collection methods can make it challenging to integrate this data into rigorous scientific analyses.

### Monitoring platforms based on coverage area

*Sargassum* monitoring requires a comprehensive approach that considers different scales of coverage, from the open ocean to the shoreline and beach. The effectiveness of each platform depends on its capacity to adapt to these scales and provide accurate, relevant data. Below, we describe the three main scales at which *Sargassum* should be monitored. Table 1 presents the classification of *Sargassum* monitoring platforms along with their respective advantages and disadvantages, based on the spatial extent they are capable of monitoring.

#### Ocean-scale monitoring

For detecting and tracking *Sargassum* in the open ocean, satellite imaging platforms represent the predominant tool. These platforms utilize sensors such as MODIS (Terra and Aqua), MSI (Sentinel), and OLI (Landsat) to provide extensive coverage, thereby enabling the observation of large *Sargassum* masses at considerable distances from the coastline (e.g., SAWS Project^28^, SIMAR^24^, and SAMTool^29^). The capacity of these platforms to capture images at regional and global scales makes them indispensable for predicting landings and planning large-scale management strategies. However, the spatial resolution of these images may be insufficient to detect smaller *Sargassum* aggregations, and cloud cover or adverse weather conditions can affect data quality.

#### Coastal line monitoring

In coastal areas, where it is crucial to monitor the imminent arrival of *Sargassum*, satellite sensors face the greatest challenges. An innovative approach to addressing this challenge is presented by^30^. This study employs values derived from the Alternative Floating Algae Index (AFAI), as calculated by the University of South Florida (USF), in an algorithm that analyzes AFAI values for pixels located at a distance of 50 km from the coastline. The algorithm calculates the difference between these values and a multi-day baseline, and subsequently classifies the risk of *Sargassum* presence into three categories: low (blue), medium (orange), and high (red). Black is used to indicate areas that lack sufficient data. The results of this methodology are published in a weekly report on the project website (NOAA SIR).

In this context, aerial drones offer a potential alternative solution. Drones can operate at low altitudes, providing high-resolution images that facilitate the identification of *Sargassum* accumulations in specific coastal areas. Their capacity to provide real-time data makes them an appropriate choice for monitoring critical coastal zones. However, their coverage area is more limited compared to that of satellite images, necessitating the use of additional technologies to cover extensive coastal stretches. Drones are particularly well suited for rapid, targeted monitoring of critical locations, such as bay entrances, high-traffic tourist areas, or sensitive nature reserves. At the time of writing, no platforms offering this type of data repository have been identified.

#### Beach monitoring

In the context of beach monitoring, direct observation platforms and in situ technologies, such as sensors installed near or on the beach, play a key role^31^. The use of beach cameras and sensors enables continuous, real-time monitoring of *Sargassum* accumulations, providing detailed data on their extent and evolution. These technologies are of considerable value for the routine management of beach cleaning and local response planning. However, their main limitation is their restricted spatial coverage, as they are constrained to the areas in which the devices are installed, rendering them less suitable for large-scale monitoring. Furthermore, maintenance of these installations can be costly and complex, particularly in coastal environments that are exposed to harsh weather conditions.

#### Community monitoring

Citizen science platforms complement monitoring efforts across all aforementioned scales by involving the local community in the collection of data. The use of mobile applications^27,32^ and community reporting networks^33^ allows the rapid gathering of information on the presence and movement of *Sargassum* in relatively wide areas of interest. While the quality and accuracy of the data may vary, they provide a valuable additional layer of observation, particularly in regions where other technologies have limited coverage or are economically infeasible. To guarantee the usefulness of this data, it is essential to implement clear data collection protocols and promote training among participants.

**Table 1:** Some advantages and disadvantages of *Sargassum* monitoring platforms as a function of spatial scale. In general terms, data sources can come from remote sensing at different scales (for instance satellite and aerial sensors) or from in situ measurements (buoys and citizen science). For a better understanding of *Sargassum* upwelling, monitoring at a range of scales and in different contexts is useful, from offshore monitoring by satellite remote sensing to in situ monitoring on the coasts and beaches.

Coverage area	Source of data	Advantages	Disadvantages
Ocean level	Satellite platforms	Extensive and regular coverage of large marine areas of *Sargassum*; allow large-scale and long-term analysis	Limited resolution for small accumulations; affected by cloud cover and weather conditions; delay in data acquisition and processing
Shoreline	Unmanned aerial vehicles (drones)	High resolution for specific monitoring; operational flexibility ; real-time data; useful for critical areas	Coverage limited to small areas; drones require trained operators and permits; flight range restricted by battery and atmospheric conditions
Beach	In situ sensors (radar, buoys, cameras)	Continuous real-time monitoring provides detailed and accurate accumulation data at specific locations	Coverage restricted to specific points; high installation and maintenance costs; vulnerability to damage
Community scale	Citizen science	Broad coverage at low cost; rapid data collection through mobile applications; involvement of the local community	Variable data quality; lack of standardization; dependent upon volunteer training and commitment

## Main *Sargassum* monitoring platforms

Although many *Sargassum* monitoring platforms are not documented in high-impact scientific journals, this does not diminish their utility and relevance in the effective monitoring of this macroalga. The absence of inclusion in such publications does not imply a lack of rigor or usefulness, as these platforms are the result of essential technological development for early warning, management, and control of *Sargassum* at various scales and contexts. Table 2 provides a summary of the main *Sargassum* monitoring platforms identified at the time of writing. Notably, the United States of America leads with four operational platforms, followed by Mexico with two, and France with one. This geographic diversity reflects the international interest in developing effective tools to monitor *Sargassum*, considering the specific needs of each affected region. One of the most important aspects to highlight is the variety of data sources employed across these platforms, which range from satellite imagery to photographs taken by the public in citizen science initiatives. This diversity of data allows information to be gathered from different perspectives and at varying levels of detail, thereby enhancing the ability to respond to the phenomenon in question. The range of sensors employed is also remarkably diverse. The most frequently utilized sensors are MODIS, OLI, MSI, and VIIRS, widely used in satellite remote sensing due to their capacity to capture information across a range of spectral bands. At the other end of the spectrum are more common sensors such as those utilized in mobile devices (CCD or CMOS technologies). While these sensors offer less precision than satellite systems, they are fundamental in citizen science schemes given their accessibility and ease of use, providing valuable validation data for the study areas. The temporal resolution of monitoring varies considerably depending on the data source. Some platforms offer daily monitoring, providing near real-time updates, while others, such as Landsat, have data collection cycles that extend up to 16 days. Spatial resolution also presents a wide range of options. Platforms designed to monitor large *Sargassum* accumulations in the ocean, such as SaWS, SATsum, or SAMTool, provide observations covering thousands of square kilometers. For example, the MODIS sensor (2330 km swat) mounted on the Terra and Aqua satellites provides a spatial resolution of approximately 1 km, while the MSI sensor (109.8 km swat) aboard Sentinel-2 satellites offers spatial resolutions ranging from 10 to 60 meters per pixel, depending on the wavelength, thereby affording a global and strategic perspective of the phenomenon. On the other hand, Planet platform images^34^ allows for a focus on areas, such as beaches or high-accumulation zones, with resolutions reaching three meters per pixel, thus providing a detailed and precise local-level analysis^35^. Nevertheless, at the time of writing, there is no known early warning website utilizing Planet platform images. The coexistence of multiple platforms addresses the need to monitor *Sargassum* from different perspectives, spatial scales, and timeframes. This ensures that the available tools can be adapted to the various scenarios under which *Sargassum* arrives from the open sea to the coastal areas, and from large-scale observations to detailed human-level analysis.

**Table 2:** Summary of the main *Sargassum* monitoring platforms identified at the time of writing this document, detailing some of their most relevant features.

Name $\setminus$ country	Data source	Operating period	Sensor	Bands or indices	Temporal $\setminus$ spatial resolution
*Sargassum* watch epicollet $\setminus$USA	Citizen science	From March 3rd 2019 to present	CCD and CMOS	RGB	Dependent upon citizen participation $\setminus \approx 2cm$
*Sargassum* watch inaturalist $\setminus$USA	Citizen science	Unknown	CCD and CMOS	RGB	Dependent upon citizen participation ∖≈2cm
NOAA coast watch ocean viewer $\setminus$ USA	Satellite images	No data	MODIS	AFAI and MSI	Almost daily $\setminus \approx 750 \textrm{m} \ to \ 1 \textrm{km}$
Satellite-based *Sargassum* watch system $\setminus$ USA	Satellite images	From February 24th, 2000	MODIS, VIIRS and OLI	AFAI, CLH, FLH, MCI, NFLH, and RGB	MODIS (almost daily $\setminus 1 \textrm{km}$), VIIRS (daily $\setminus 375 \textrm{m}$) and Landsat-OLI (16 days $\setminus$ 30 m)
Collective view $\setminus$Mexico	Citizen science	From Sept. 14th, 2019 to July 2021	CCD and CMOS	RGB	Dependent upon citizen participation $\setminus \approx \textrm{cm}$
SIMAR-*Sargassum* satellite early warning system $\setminus$Mexico	Satellite images	From January 2010	MODIS	AFAI	Almost daily $\setminus 1 \textrm{km}$
SAMTool- *Sargassum* detection $\setminus$ France	Satellite images	Unknown	MODIS, OLCI MSI, and OLI	AFAI	MODIS (almost daily $\setminus 1 \textrm{km}$), and Landsat - OLI (16 days $\setminus 30 \textrm{m}$)

### *Sargassum* monitoring platforms for the Mexican Caribbean

The need to implement novel *Sargassum* monitoring platforms in the Mexican Caribbean is predicated on the complexity and dynamics of this phenomenon, as well as the diversity of management and mitigation objectives specific to this region. *Sargassum* exhibits considerable spatial and temporal variability in the Mexican Caribbean, with fluctuations in distribution and movement that can vary significantly along the coast. As such, a single monitoring platform would be inadequate for capturing all the crucial aspects of the behavior of these macroalgae in this region. Large-scale observations (synoptic and mesoscale), such as those obtained by satellites, are essential to identify general patterns of *Sargassum* arrival in the Mexican Caribbean region. However, these tools lack the resolution required to detect smaller details near beaches, where the ecological and socioeconomic impacts are more pronounced, for this purpose, the pixel size less than or equal to one kilometer is required. For example, coastal communities and the tourism sector in Quintana Roo are strongly impacted by the ability to anticipate and mitigate the arrival of *Sargassum*, underscoring the importance of monitoring platforms that can provide data at finer scales. Current platforms present limitations in terms of the spatial and temporal resolution required for effective *Sargassum* management along the Mexican Caribbean coasts. There are also challenges inherent in the existing predictive models, as they do not fully reflect the complex interactions between ocean currents, winds, and local geographic and bathymetric features that influence *Sargassum* accumulation and distribution in this region. This can lead to inaccurate forecasts and ineffective upwelling management responses. In response to the above, this study presents an innovative *Sargassum* monitoring platform for the Mexican Caribbean, which incorporates images from the MSI and OLI sensors of the Sentinel 2 and Landsat 8-9 platforms, respectively, allowing monitoring with a temporal resolution of 5 days, at a spatial scale of 20 and 30 m per pixel, thus improving the spatial resolution of the current observations of the Mexican Caribbean coastal zone. In addition, the proposed platform is designed to be highly adaptive and capable of integrating data from diverse sources and adjusting rapidly. This is crucial in a climate change context, where the frequency and magnitude of *Sargassum* upwelling in the Mexican Caribbean is constantly evolving. This proposal contributes toward obtaining an increasingly reliable early warning system, which is fundamental for the planning and execution of more effective mitigation measures.

### LANOT *Sargassum* monitoring platform

**Figure 1:**
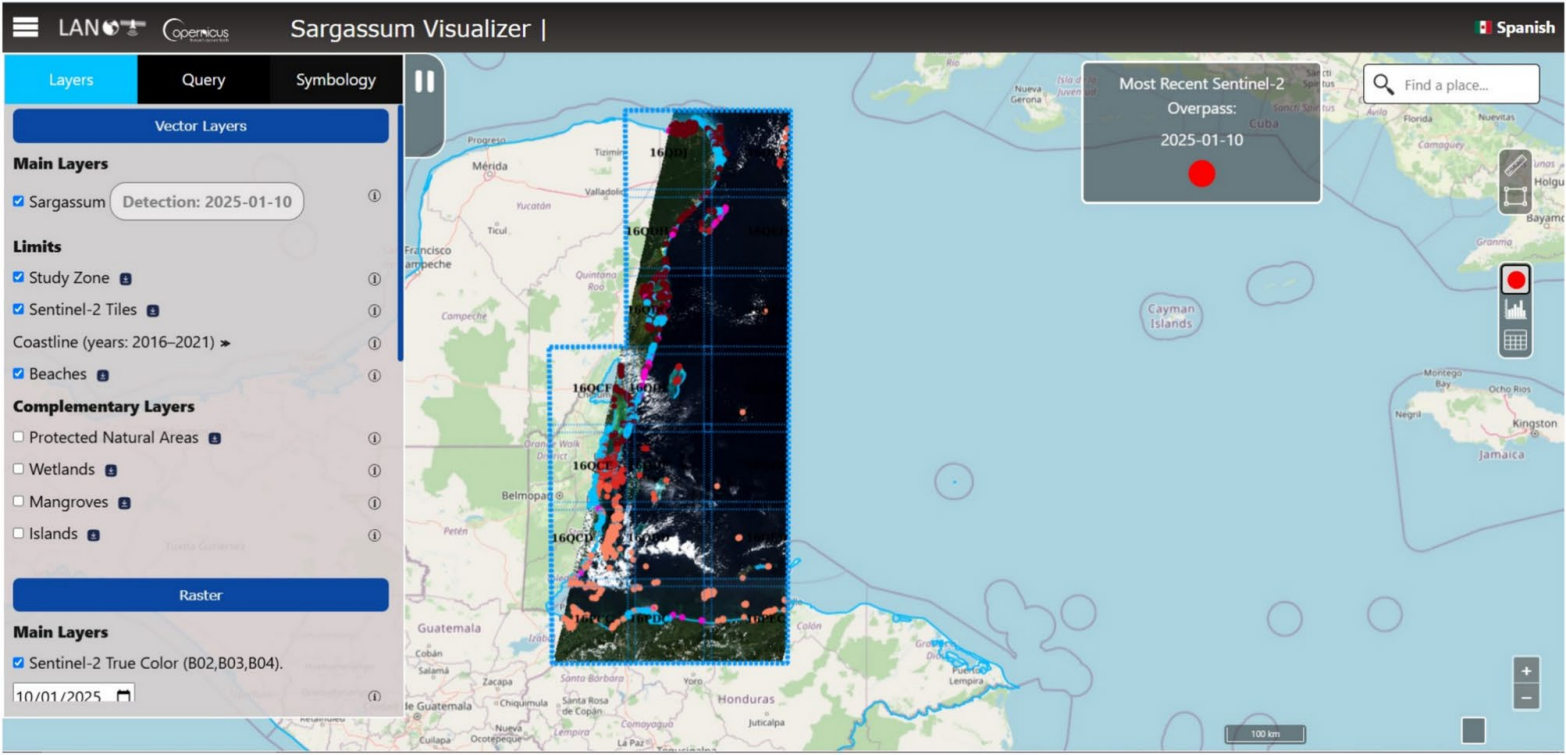
LANOT platform (http://www.lanot.unam.mx/home/sargazo_en/visualizador_en/) main window. The study area includes the Caribbean Sea of Mexico, Belize, Guatemala, and Honduras. The main menu offers three subsections from which to select the desired settings and is displayed on the left side. The central part shows the main map, in which the parameters set in the menu are depicted. Sentinel-2 imagery used by the platform corresponds to those assigned open access policies by ESA^37^.

The National Laboratory for Earth Observation (LANOT) of the Geography Institute of the National Autonomous University of Mexico (IG-UNAM) has developed and implemented an innovative web platform for monitoring *Sargassum* in the Mexican Caribbean region^36^. This visualizer, designed by LANOT (hereafter referred to as LANOT platform), represents a significant contribution toward the understanding and management of the impact of this macroalga in the region. The platform has become a comprehensive reference tool, providing key information on the presence and distribution of *Sargassum* along the coasts of the Mexican Caribbean, as well as in other affected regions, including Belize, Guatemala, and Honduras (Fig. 1).

The LANOT platform implements techniques already known in the state-of-the-art for the visualization of *Sargassum*. On the one hand, it implements the FAI (Floating Algae Index) on Landsat images and, on the other, it applies photointerpretation to Sentinel-2 images.

Visual inspection has been used in MODIS images (best spatial resolution 250 - 500m) for the identification of floating spots associated with *Enteromorpha prolifera* in the western North Atlantic and *Sargassum* in the Gulf of Mexico^19^, because the presence of macroalgae in these regions is known for the dates of observation, the results of the photointerpretation have coincided with the observations of the phenomenon in these regions. On the other hand, the morphology of *Sargassum* slicks helps in their visual identification^20^, and the contrast can be set by interactive color stretching^19^. Therefore, photointerpretation of MODIS images has aided the identification of floating algae even in clear water.

In this context, LANOT-qualified personnel conducted a visual inspection of Sentinel-2 imagery (best spatial resolution 10-60 m) and observed that for the study area, the band’s combinations (expression 1) allow a useful observation of the *Sargassum* slicks. The analyzed imagery corresponds to dates on which there are records of *Sargassum* presence on the coasts and beaches of the region.

Therefore, the photointerpretation work allowed the development of a filtering expression for the detection of *Sargassum* in the Mexican Caribbean. This expression (1) takes advantage of the spectral properties of the Sentinel-2 sensor bands (from 665 nm in the visible red region to 1610 nm in the near-infrared and shortwave). The integration of these bands allows us to distinguish the *Sargassum* from the background noise.

1 $$\begin{aligned} (b8A<0.07) and (b04<0.1) and (b11<0.05) and (b04<b8A) and (b04<b08) \end{aligned}$$

The design of this filtering expression is based on a series of coefficients that are carefully calibrated for the study region. This calibration process considered optical and physical factors inherent to the Mexican Caribbean environment, including the light scattering and absorption characteristics of coastal waters, as well as the particular reflective properties of *Sargassum*.

The functionalities of the platform include the provision of updated maps and graphics, which illustrate the occupied area and georeferenced location of the *Sargassum* rafts, along with other detailed quantitative, qualitative, and geospatial data. These tools have been instrumental in the analysis of the phenomenon, providing researchers and decision-makers with information for planning and implementing mitigation measures. Among the most outstanding resources of the geo-portal is a collection of satellite images from May 2016 up to the present from the Sentinel-2 platform, which provides continuous visual coverage of *Sargassum* along the coasts of Mexico, Belize, Guatemala, and part of Honduras. The portal also includes interactive tools that allow the measurement of distances, calculation of affected areas, and visualization of dynamic predictive models of *Sargassum* trajectory and behavior. This facilitates a comprehensive analysis of the phenomenon and supports data-driven decision-making.

An outstanding feature of LANOT’s geo-portal is its capacity to integrate multiple data sources and present these in an accessible and usable manner, thus facilitating the understanding of *Sargassum* dynamics in the Mexican Caribbean. This comprehensive approach allows the platform to provide usable information to support the development of strategies that contribute to preventing and mitigating the negative impacts of *Sargassum* on navigation, tourism, fisheries, and coastal ecosystems. In this context, the LANOT’s geo-portal has become a reference for *Sargassum* monitoring in the region, providing a baseline for developing new observation tools to overcome current limitations and improve the capacity to respond to this ever-changing phenomenon. The *Sargassum* viewer has three main sections: layers, query, and symbology. These provide usable information for analyzing *Sargassum* with vector and raster data. In the layers section, two types of resources are presented: vector and raster. In the vector format, the layer “*Sargassum*” provides polygons of geospatial data, representing the concentrations detected by images produced by the multispectral sensor (MSI) aboard the Sentinel-2 satellites. This capability enables regular observations of the Mexican Caribbean and adjacent countries, with a five-day temporal resolution from 2017 to the present. Vector data available in the viewer have provided an accurate delineation of *Sargassum* concentrations since 2017. This information allows a detailed temporal and spatial analysis of the distribution of these macroalgae. Each polygon in the *Sargassum* layer contains an attribute table with key data, such as a unique identifier, the date of image acquisition, the processed tile, the area of the polygon in square kilometers, the distance of the polygon from the coast (represented by a color scale), and the type of detection location (ocean, beach, or other seaweed). These attributes are useful for mapping coastal areas affected by *Sargassum*, facilitating effective response planning and waste management in those areas.

To differentiate stranded *Sargassum* from coastal vegetation in the development of our monitoring platform, we implemented an approach based on multi-temporal shoreline characterization and cover classification using Sentinel-2 imagery at 10-meter resolution. First, we generated annual shorelines from 2018 to 2022 to delimit beach areas, identifying those areas devoid of vegetation through dry and rainy season analysis, which allowed for more accurate segmentation. Only sandy beaches were considered to avoid confusion with mixed coastal ecosystems. This procedure ensures that the detection of *Sargassum* on beaches is based on recent changes in areas previously classified as vegetation-free, minimizing interference with stable coastal vegetation formations.

**Figure 2:**
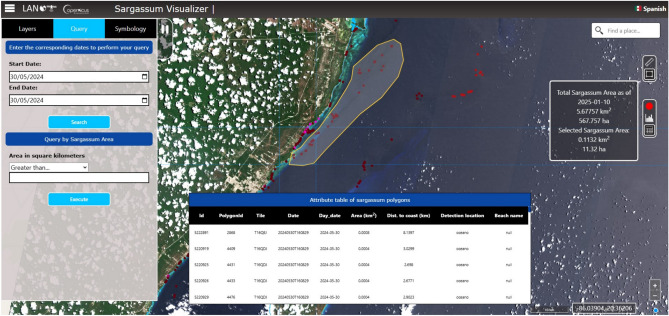
LANOT platform (http://www.lanot.unam.mx/home/sargazo_en/visualizador_en/) main menu with the different sections: Layers, Query, and Symbology (left side window). The tool for the selection of areas of interest allows the display of dialog windows and information regarding the attributes of the polygon constructed by the user. The polygon is located in the center of the image, and the attribute table is presented at the bottom. Sentinel-2 imagery used by the platform corresponds to those assigned open access policies by ESA^37^.

The *Sargassum* viewer also has a toolbar that enables filtering or highlighting the aforementioned data. It also has a ruler that allows the measurement of distances between two points and offers the option to quantify the area of a polygon of interest drawn by the user. A further tool, the area selector, enables a rectangle or polygon to be drawn on the map. This generates two dialog boxes: the first shows the total surface of *Sargassum* in the study area and the specific area of *Sargassum* within the selected polygon; the second displays the table of attributes of the chosen *Sargassum* polygons (Fig. 2).

**Figure 3:**
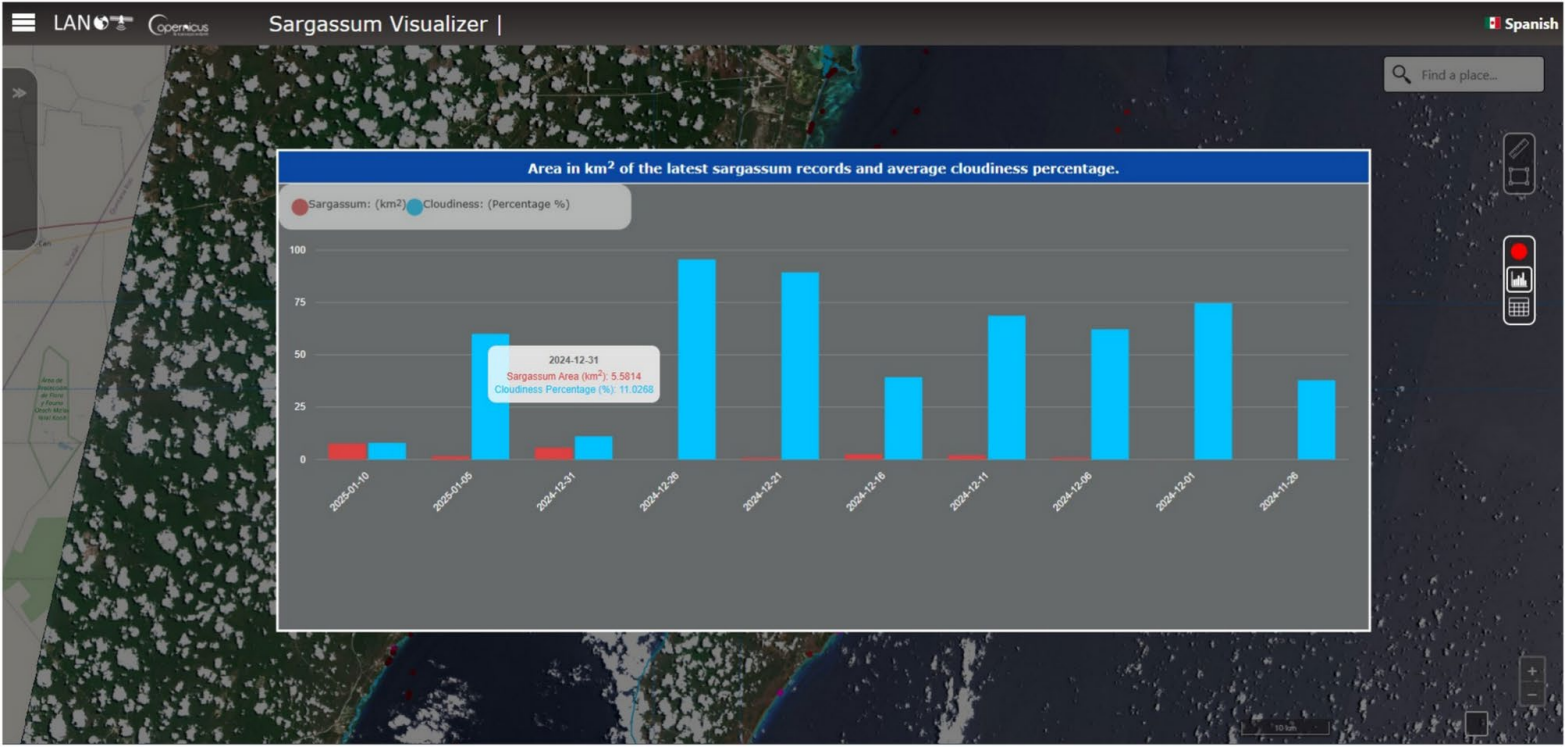
LANOT platform (http://www.lanot.unam.mx/home/sargazo_en/visualizador_en/) statistics window of the last ten steps of the sweep of the 18 processed satellite tiles, providing information regarding the total amount of *Sargassum* detected, and the percentage of clouds for the entire study area, which facilitates the correlation between the observation of *Sargassum* and the cloud conditions. Sentinel-2 imagery used by the platform corresponds to those assigned open access policies by ESA^37^.

The viewer also has a graph section that allows consultation of the ten most recent satellite passes in the study region, providing information about the detected area (km$^2$) of *Sargassum* and the percentage of clouds in the 18 processed tiles. These facilitate the correlation between *Sargassum* detection and atmospheric conditions during the capture of the satellite images (Fig. 3). Moreover, the attribute table allows users to access detailed information about each detected *Sargassum* polygon, for instance, the name of the tile in which the polygon was detected, the area in kmÂ², the type of detection site (ocean, beach, or other seaweed), and the date and time of image acquisition. In cases where the names of the beaches near the *Sargassum* detection area are recorded, these will be displayed in the attribute table.

The platform includes other relevant layers, grouped under the “límites” section, covering the study area (713 km x 309 km), Sentinel-2 tiles, coastline, and beaches. The study area is delimited by 18 Sentinel-2 sweep grid tiles, identified with the following nomenclature: T16QDJ, T16QEJ, T16QDH, T16QEH, T16QDG, T16QEG, T16QDF, T16QEF, T16QCF, T16QCE, T16QDE, T16QEE, T16QCD, T16QDD, T16QED, T16PCC, T16PDC, and T16PEC. These tiles represent $109.8 \times 109.8 \ \textrm{km}^2$ granules in UTM/WGS84 projection^38^, which is the coordinate system used in the geo-portal’s geospatial products. The coastline and beaches layer, also part of the “límites” section, was developed with Sentinel-2 data. For its creation, 32 level 2A images per year were used, selected from the Copernicus portal^39^. These had a percentage of cloudiness below 20%, given the high cloudiness characteristic of the region during most of the year. Within the vector section, additional layers are included to analyze the presence and distribution of *Sargassum* in different parts of the study area. These layers include natural protected areas, wetlands, mangroves, and islands. The layer of Federal Protected Natural Areas of the Mexican Republic contains spatial data according to the decrees published in the Official Journal of the Federation, produced with Geographic Information Systems tools provided by CONANP^40^. Potential wetlands are delimited with models based on ecological characteristics such as vegetation, soil, water, and slope, which serve to identify areas with the capacity to host wetlands^41^. The mangrove layer shows the distribution and extent of these ecosystems for the year 2020, using a classification method derived from the 2015 land use and vegetation map, with a particular focus on coastal mangrove areas^42^. Finally, the layer of islands extracted from the cartographic collection “Entidades Federativas de la República Mexicana” is relevant due to its touristic and ecological importance in the region^43^.

Regarding the raster section, layers from Sentinel-2 and Landsat-(8-9) are included. The first is a true-color composite image from Sentinel-2, using the combination of RGB bands: red band 4 (0.665 $\upmu$m), green band 3 (0.560 $\upmu$m), and blue band 2 (0.490 $\upmu$m) to create an image that reflects the Earth’s surface as it would be seen from space in the visible spectrum. The second Sentinel-2 layer is a false-color image, using the combination of bands: 8A (0.865 $\upmu$m), 5 (0.705 $\upmu$m), and 4 (0.665 $\upmu$m), which highlights floating vegetation, such as *Sargassum*, in bright red or green tones while water appears in dark blue tones, providing a clear visual contrast.

**Figure 4:**
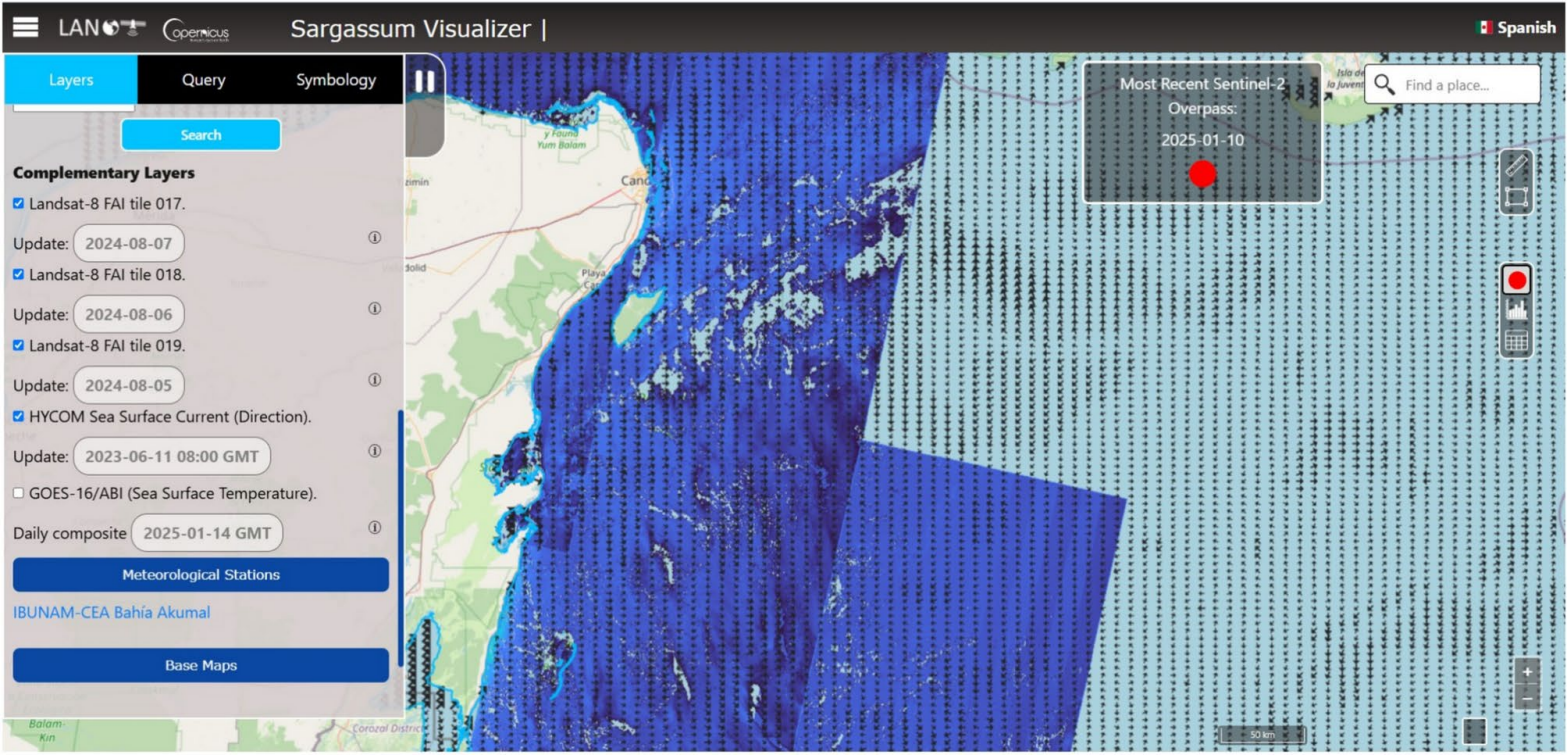
LANOT platform (http://www.lanot.unam.mx/home/sargazo_en/visualizador_en/) raster window, here are possible setting parameters using the pop-up menu located on the left side of the main window. The FAI index (calculated using Landsat-8 imagery), and HYCOM vector layers, are displayed in the central right side of the window. Landsat imagery used by the platform corresponds to those assigned open access policies by NASA^44^.

The third layer available corresponds to Landsat-8 (Fig. 4), using the Floating Algae Index (FAI), an algorithm designed to measure the intensity of the red edge of floating vegetation. FAI has been validated on various sensors such as MODIS, VIIRS, MSI, and OLI, and is effective for *Sargassum* detection in areas that present high variability in water reflectance^20,30,45^. FAI values, ranging from $1.79 \times 10^{-4}$ to 0.044, exhibit a monotonic relationship with *Sargassum* cover density. Finally, two additional layers are included: the HYCOM (HYbrid Coordinate Ocean Model), which simulates the general ocean circulation with a temporal resolution of three hours^46^, and the Sea Surface Temperature layer, calculated from images of the ABI sensor of the GOES-16 satellite. This satellite provides information every 10 minutes, generating a daily composite that averages the temperature per pixel and eliminates clouds. This data is downloaded directly from LANOT.

**Figure 5:**
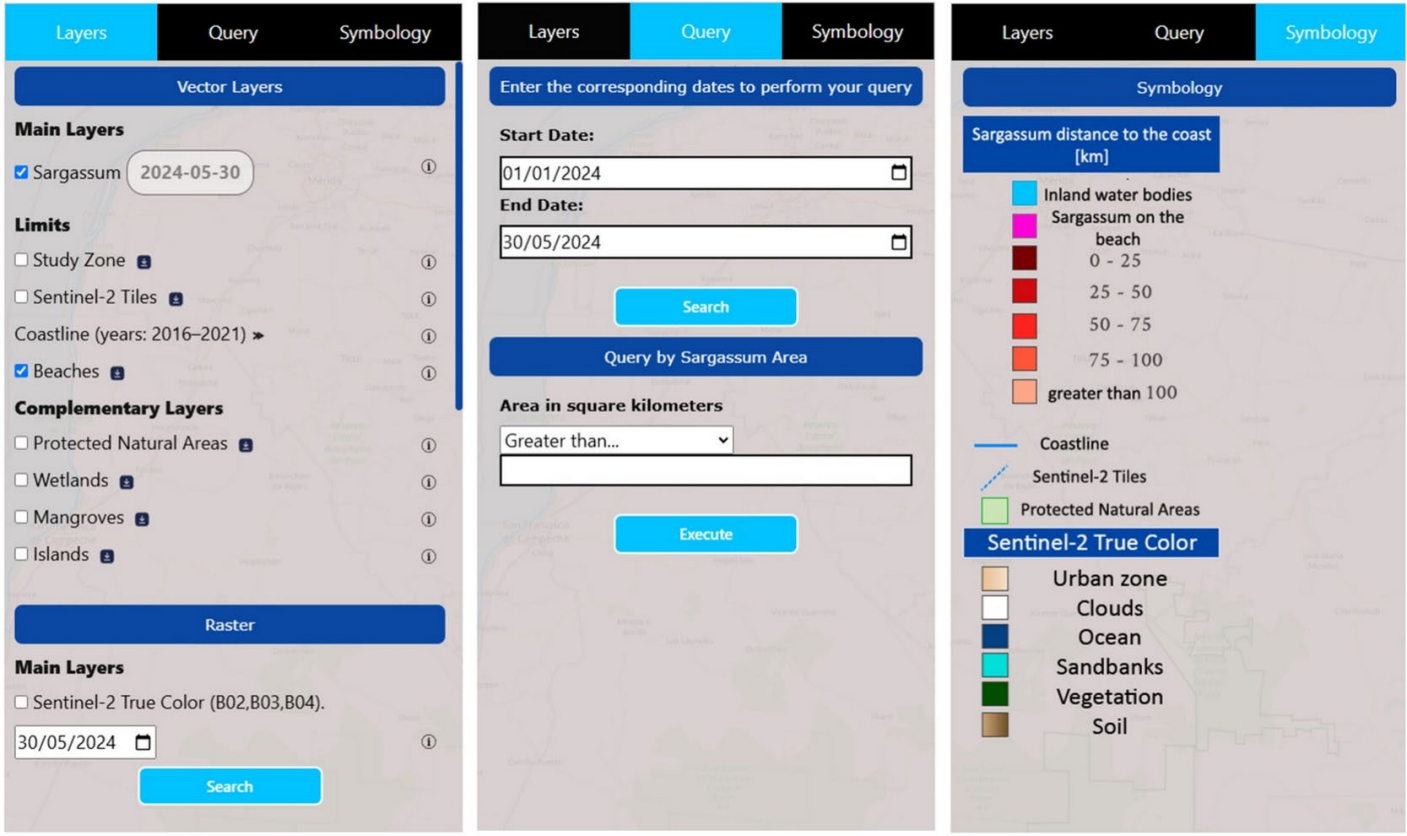
LANOT platform submenu details and parameters that can be adjusted in the subsections “Layers” , “Query” , and “Symbology”.

The second section of the geo-portal is for consultation. In this interactive section, users can enter specific criteria to filter the information according to time period and visualize the data available on the site. The LANOT-viewer provides updated information on the Mexican Caribbean from the most recent Sentinel-2 sweep, with updates every five days. In the case of Landsat, only the last sweep made by this satellite in the study area is available. It is also possible to perform queries by area using operators such as greater than or equal to, less than or equal to, and equal to. For instance, if it is desired to visualize floating rafts detected by the algorithm with an area greater than five $km^2$ on a specific date on which Sentinel-2 provided information or within a certain period, the display will show the polygons corresponding to those criteria. Finally, the section “Simbología” contains the icons and colors representing the data activated in the layers section, which improves the understanding and visualization of the geographic information displayed on the map. The information summary of the sections “Capas”, “Consulta”, and “Simbología” is depicted in Fig. 5.

The information shown in the interactive map^36^ is of great value since it allows the superimposition of different layers to perform a visual interpretation of *Sargassum* distribution in the Mexican Caribbean. In response to the need to offer these data in distinct formats, the portal allows downloads of all available information from the end of 2017 to the most recent image. The available data present various levels of processing. Compressed images, processed from L1C to L2A level using Sen2Cor software 2024^47^, can be downloaded, as well as the final *Sargassum* polygons obtained with the algorithm developed by LANOT, in GeoJSON format. The use of Sen2Cor^47^ for atmospheric correction within our platform is based on its specific design for processing Sentinel-2 images, its ability to differentiate between land and water surfaces, and its efficient integration into automated workflows. One of its main advantages is the generation of the Scene Classification Layer (SCL), which provides indispensable cloud masks to minimize false positives in *Sargassum* detection, an aspect that ACOLITE^48^ and C2RCC^49^ do not offer. In addition, previous studies, such as that of Cao et al. (2021)^50^, have shown that the application of Sen2Cor^47^ in the detection of floating algae has achieved significant correlations ($R^2 = 0.85$) in the spectral reflectance of *Sargassum*, validating its effectiveness for this type of monitoring. Likewise, research such as that of Li et al. (2023)^51^ has successfully employed Sen2Cor^47^ in the classification of floating algae, achieving accuracies of 97% using spectral indices derived after atmospheric correction.

**Figure 6:**
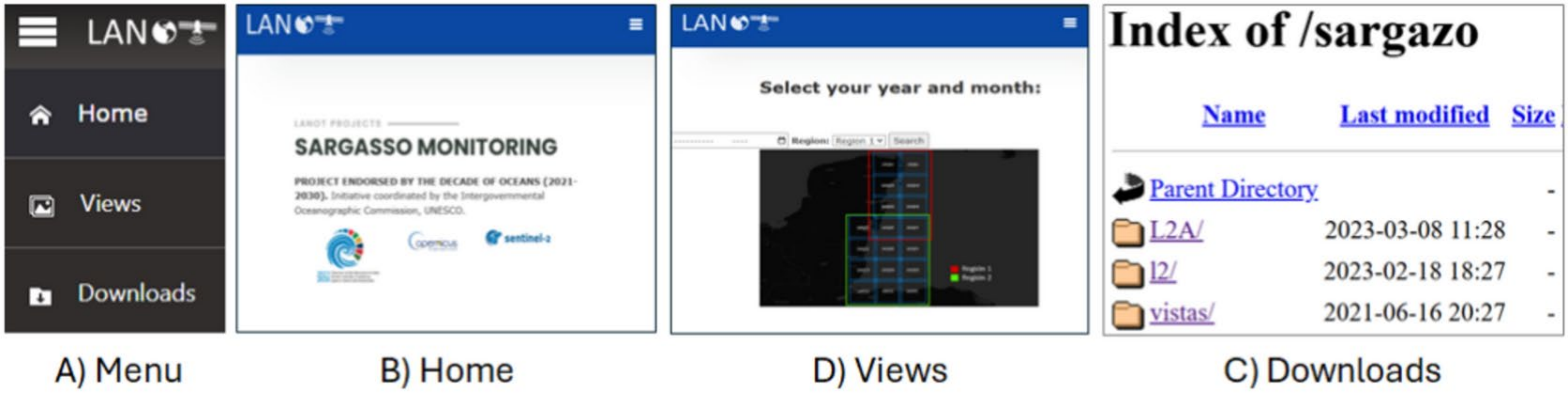
Detail of the LANOT platform main menu information. (**A**) Main menu, (**B**) “Home” button linking to the LANOT project home page^53^, (**C**) “Views” button linking to the search page, to select and download *Sargassum* imagery (PNG files)^52^, and (**D**) “Downloads” button linking to the website for downloading imagery (geotiff, geojson, and png files).

In addition, general views of the study are provided in a true-color RGB composite, with the overlay of the *Sargassum* rafts in PNG format^52^. The files are organized according to Sentinel-2 product and tile and are accessible through the download section (Fig. 6). The importance of the geo-portal of LANOT lies in its ability to integrate multiple types of data and present them in an accessible and useful manner, facilitating the understanding of *Sargassum* dynamics in the Mexican Caribbean. This holistic approach has established the platform as a reference in *Sargassum* monitoring for the region, providing information vital to the development of effective solutions for the prevention and mitigation of the impacts of this floating alga on navigation, tourism, fisheries, and coastal ecosystems in the region. In this context, the geo-portal of LANOT has established a standard for *Sargassum* monitoring in the Mexican Caribbean and provides a foundation baseline on which novel monitoring tools can be designed to address current limitations and improve responsiveness to this dynamic phenomenon.

The LANOT platform development was based on a modular architecture that integrates multiple technologies, selected according to the needs of each stage of the workflow. In the first phase, corresponding to image download and preprocessing, Python 3.9.2^54^ was used as the base language, taking advantage of its ecosystem of libraries for geospatial data processing, and Django: 3.2.8^55^ as a framework. The retrieval of L1C-level Sentinel-2 imagery was performed using Copernicus OData REST API 1.0^56^, with an automated script that executes periodic queries. Subsequently, the conversion to L2A level and atmospheric correction was performed by Sen2Cor 2.12^47^, a tool developed by the European Space Agency (ESA). In this phase, Python^54^ is also used to manage pixel classification using the Scene Classification Layer (SCL), facilitating the identification of relevant areas for *Sargassum* detection. The second and third stages include detection, storage, and visualization. Python^54^ is still used for the application of segmentation algorithms, entropy filtering, and denoising by thresholding spectral values. The conversion of classified pixels into vectors was performed with GDAL 3.2.2^57^ and Shapely 2.0.1^58^, and the results were stored in a spatial database in PostgreSQL/PostGIS 11.3/2.5^59^. The platform is integrated into a server where GeoServer 2.15.2^60^ provides the data in WMS format, allowing visualization through a web interface based on Django (Python, MVC), Openlayers 6.15.1^61^, and JavaScript, with CSS managed layout.

## Discussion

The products and methodologies provided by the USF SaWS are benchmarks for *Sargassum* observation in the ocean. Consequently, they are employed in most of the efforts made by other institutions and countries in the region to address the challenge posed by the extensive *Sargassum* accumulations on their coasts and beaches. Although ocean observation presents challenges, such as improving the spatial resolution of its observations, the resulting currently available information has been useful for practically all the affected countries. We therefore believe that the most pressing current challenge lies in the design and deployment of early warning platforms that can allow the observation and monitoring of *Sargassum* in coastal waters and beaches. In this context, proposals such as the NOAA CoastWatch Ocean Viewer and LANOT have focused on coastal monitoring. On the other hand, the proposals of *Sargassum* Watch and Collective View have demonstrated that citizen monitoring can supplement remote sensing by monitoring particular areas of interest. Table 3 shows 12 characteristics that could help users select which platform is most appropriate for their needs. The users may be academics or decision-makers, both of which will have very particular needs. A platform may therefore be of greater or lesser demand, depending on the need and location of the user. This is shown as an example merely to identify the different aspects that a *Sargassum* monitoring platform may present. For users who need to perform some type of analysis, it is useful to have downloadable files for on-demand use. For decision-makers, however, the most desirable thing is to have unified and clear information, and it is therefore important to have periodic bulletins with summaries and outlines of the most relevant information covering the period and area of interest.

**Table 3:** Summary of some features of *Sargassum* observation and monitoring platforms identified at the time of writing this document. *SAMTool is a paid platform, so additional services are unknown.

Platform name	Ocean info.	Coastal info.	Beach info.	Vector files	Raster files	KLM files	Length raft	Area raft	Attribute table	Animation	Bulletin	Forecast
*Sargassum* Watch (epicolet)			$\checkmark$		$\checkmark$							
*Sargassum* Watch (iNaturalist)			$\checkmark$		$\checkmark$							
Collective view			$\checkmark$									
Satellite-based *Sargassum* Watch System	$\checkmark$					$\checkmark$				$\checkmark$	$\checkmark$	$\checkmark$
NOAA-CoastWatch Ocean Viewer	$\checkmark$	$\checkmark$				$\checkmark$					$\checkmark$	
SIMAR- SATsum	$\checkmark$				$\checkmark$	$\checkmark$	$\checkmark$				$\checkmark$	
CARICOOS	$\checkmark$											$\checkmark$
Visualizador sargazo LANOT		$\checkmark$			$\checkmark$		$\checkmark$	$\checkmark$	$\checkmark$			
SAMTool	$\checkmark$											$\checkmark$

However, to provide relevant information that facilitates informed decision-making in society, significant efforts that summarize information from monitoring platforms have been published as bulletins. Most of these bulletins have main inputs to the information provided by the University of South Florida (USF) through the Optical Oceanography Laboratory. Some of the bulletins available at the time of this writing are as follows: USF Monthly *Sargassum* Bulletin^28^, NOAA-USF: Experimental Weekly *Sargassum* Inundation Risk (SIR v1.3)^62^, *Sargassum* Surveillance Bulletin for Guadeloupe^63^, and *Sargassum* Sub-Regional Outlook Bulletin^64^. A summary of some of the characteristics of the *Sargassum* bulletins available at the time of writing this article is presented in the table 4.

**Table 4:** Summary of some of the characteristics of the *Sargassum* bulletins available at the time of writing this article.

Name bulletin $\setminus$ institution	Periodicity	Coverage	Products
USF Monthly *Sargassum* Bulletin $\setminus$ University of South of Florida	Monthly from January 2018 to date	Along the Great Atlantic *Sargassum* Belt	Average Sargassum abundance for the month along the Great Atlantic *Sargassum* Belt, and its comparison with the months of previous years
NOAA-USF: Experimental Weekly *Sargassum* Inundation Risk (SIR v1.3) $\setminus$ National Oceanic and Atmospheric Administration (NOAA), and University of South Florida (USF)	Every week from July 2019 to the present	Along the coastal zones of Caribean sea	By analyzing the AFAI values of each coastal pixel and calculating the difference between these values and a multi-day baseline, the risk of *Sargassum* presence is classified into three categories: low, medium and high
*Sargassum* Surveillance Bulletin for Guadeloupe $\setminus$ Direction de l’Environnement, de l’Aménagement et du Logemen (DEAL) de Guadeloupe	Weekly, only from January to December 2020	Regional information around Island Guadeloupe	Map and estimate of *Sargassum* only in Guadeloupe’s coasts HTML and PDF files
*Sargassum* Sub-Regional Outlook Bulletin $\setminus$ Centre for Resource Management and Environment Studies and University of the West Indies at Cave Hill (UWI)	Quarterly, 23 bulletins from October 2019 to the present	Eastern Caribbean islands	It provides the abundance of *Sargassum* in a 7-day period, as well as simplified comments on scientific and technological developments for the better understanding of stakeholders

The LANOT platform offers a significant capacity to integrate different types of geospatial data, allowing scientists, managers, and decision-makers to obtain a comprehensive view of the phenomenon. However, it is important to consider both its strengths and areas for improvement. One of the main advantages of this platform is its ability to integrate data and sensor images from Sentinel-2 and Landsat-(8-9) satellites. These satellite platforms provide multispectral imagery that allows the detection of *Sargassum* using near-infrared bands to capture variations in reflectance, an essential attribute in the identification of algae in the ocean. This multispectral approach has proven to be very efficient in monitoring large areas and providing detailed geospatial information, making it a crucial tool for studies at various scales. A unique feature of the *Sargassum* monitoring platform developed by LANOT is its ability to work interactively, allowing users to measure distances and areas of selected *Sargassum* rafts. This functionality provides an invaluable tool for *Sargassum* research and management, as it facilitates analysis of the distribution and extent of the macroalgae. Users can plot distances between different points of interest, calculate affected areas, and generate accurate spatial metrics essential for mitigation action planning. This geospatial interaction capability, combined with satellite data visualization, provides a significant advantage over other platforms, enabling a faster and more informed response at critical times when *Sargassum* impacts the coasts. Another feature of the LANOT platform is its ability to provide up-to-date data with a five-day temporal resolution, together with a predictive model of *Sargassum* dynamics. Periodic monitoring employing this review interval, and the dynamic forecast dispersion and landfall map of *Sargassum* rafts, ensures that users have recent information on which to base their decision-making. This frequency is crucial for managing the arrival of *Sargassum* on the coasts since it allows for efficient planning of collection and mitigation operations, minimizing the impact on affected tourist and fishing areas.

Despite these benefits, the platform also has limitations that must be addressed to improve its effectiveness. For instance, an important improvement would be the generation of periodic bulletins, as well as the implementation of customizable subscription schemes. We believe that this would help to establish communication channels between the platform and its users, promoting a dissemination of knowledge that adapts to the needs of different audiences. In addition to contributing to the dissemination of technical information and platform updates, these bulletins would also support the education of society regarding *Sargassum* dynamics and the latest mitigation strategies. This improvement would be especially valuable in terms of integrating the community into proactive management, allowing a coordinated and efficient response to critical events.

On the other hand, incorporation of a mobile application would expand the reach and accessibility of the platform, allowing users to consult maps, receive personalized alerts, and report data in a more efficient manner. In addition, the integration of automatic *Sargassum* raft classification algorithms, based on criteria such as size, density, and potential impact, would facilitate the prioritization of efforts. It would also be critical to link the platform with regional crisis management or environmental protection systems, ensuring that updated data can be employed to coordinate immediate and effective responses.

In the same sense, the mobile application could support the integration of citizen science schemes to provide in situ information, allowing local users to report *Sargassum* sightings through mobile devices, which would strengthen the platform’s database and promote the participation of coastal communities. Moreover, the inclusion of ecological and economic impact indicators would help assess the potential effects of *Sargassum*, from biodiversity loss and coral reef damage to the costs associated with cleanup efforts, thus providing a more comprehensive view for decision-making.

Although the spatial resolution of Sentinel-2 imagery is adequate for regional studies, with coverage of $109.8 \ \times \ 109.8 \ km^2$ per image and 20 to 60 m per pixel, this resolution may not be sufficient in situations that require more detailed analysis, such as the case of *Sargassum* accumulation in specific areas of beaches. In such instances, finer spatial resolution imagery would be desirable to identify small deposits of *Sargassum* and perform more accurate monitoring at the local level. High spatial resolution sensors, such as those from commercial satellites like the Planet constellation^34^ or unmanned aerial vehicles (UAV), could be a complementary option.

In summary, citizen-operated mobile devices and drones could complement satellite coverage in areas that are difficult to monitor, or during periods of high cloud cover. This would allow for greater data density and thus help improve the accuracy of *Sargassum* detection, especially in the most vulnerable coastal areas. This information contributes to users defining specific parameters, such as the size of the rafts and their proximity to the coast, and could provide critical information for coastal communities, environmental management institutions, and tourism-dependent economic sectors with which to anticipate and prepare mitigation strategies tailored to their particular contexts.

On the other hand, although the five-day interval is adequate for many uses, there are periods when cloud cover can interfere with the acquisition of optical images. This is an inherent limitation of passive satellite systems that rely on sunlight, such as Sentinel-2. In regions such as the Caribbean where cloud cover is frequent, especially during the rainy season, the consequent lack of satellite data at certain times could affect the five-day monitoring capability. Some alternatives to address this limitation are integrating imagery from the Planet platform to improve the spatial accuracy of monitoring, images from the Sentinel-3 platform to enhance the frequency of optical data acquisition, or incorporating Synthetic Aperture Radar (SAR), which provides images that are less affected by clouds^65^.

These improvements seek to optimize the platform’s capacity to provide more detailed and timely information on the presence and movement of *Sargassum* in the Mexican Caribbean, increasing its usefulness as a tool for decision-making and environmental management. Although these sensors are not currently available within the platform, it is important to note that their absence does not compromise the current functioning and utility of the system. The LANOT platform already provides reliable and up-to-date geospatial information using Sentinel-2 and Landsat-8/9 satellite imagery, which enables monitoring. These capabilities have proven sufficient to meet the primary monitoring objectives, and any future enhancements will be a complement to enhance its performance, rather than an essential condition for its current operation.

Finally, a potential enhancement to the platform is the integration of more advanced predictive models that not only show the current location of *Sargassum* but also provide detailed predictions of future *Sargassum* behavior based on meteorological and oceanographic data. Although the platform already includes predictive models, as described above^53^, these could be strengthened using artificial intelligence and machine learning, allowing users to more accurately anticipate *Sargassum* movement and optimize the response in areas of highest risk. Finally, a summary of strengths and areas for improvement by LANOT platform are shown in Table 5.

**Table 5:** Advantages, disadvantages, and areas of opportunity presented by LANOT platform.

Aspect	Advantage	Disadvantage	Opportunities
Satellite data	Integration of Sentinel-2 and Landsat-8/9 data, providing accurate and high-quality multispectral imagery	Reliance on optical data, affected by cloud cover	Incorporation of radar sensors (SAR) that are unaffected by weather conditions
Temporal resolution	Monitoring with a review frequency of 5 days, allowing for regular updating of data	Cloudy conditions may prevent image acquisition at critical times	Improve update frequency by combining satellite data with real-time citizen science data
Spatial resolution	Adequate resolution for regional monitoring ($\approx 109.8 \times 109.8 \ \textrm{km}^2$) per granule, allowing a broad analysis of the affected areas	Limited resolution for monitoring specific areas or small accumulations on beaches	Incorporate higher resolution imagery (commercial satellites or drones) for more accurate local surveillance
Forecasting models	Includes dynamic models to predict the trajectory and behavior of *Sargassum*	Current models may be limited in accuracy	Incorporate predictive models through machine learning
Citizen science	Potential to incorporate data collected by citizens using mobile devices and drones	The use of citizen science to complement satellite data has yet to be fully utilized	Develop more effective methodologies to integrate real-time citizen data and improve the density and accuracy of monitoring
Use of information	Provides detailed geospatial and temporal data that facilitates decision-making and mitigation action planning	High specialization of the data can be difficult to interpret without specific technical training	Improve accessibility and understanding of data for users with different levels of expertise

## Conclusions

In light of the considerable strandings of pelagic *Sargassum* in countries of the Greater Caribbean, the establishment of a “last mile” monitoring system would be of great value. Such a system would facilitate the accurate estimation of the quantity of *Sargassum* that is likely to reach their shores and beaches. Such a monitoring system would be extremely valuable to mitigate the ecological, economic, and social impacts associated with the massive accumulation of these macroalgae. The impact on coastal ecosystems and economic activities such as fishing and tourism justifies the need to develop *Sargassum* monitoring platforms that contribute to effectively predicting and managing the arrival of *Sargassum*. In this sense, the monitoring platform developed by LANOT has emerged as a key tool to address this challenge in the Mexican Caribbean region. Currently, the platform offers information in both Spanish and English languages, and future versions of the platform will have menus in French, in order to expand the platform’s services to users from other Caribbean countries. The LANOT platform contains information from the Caribbean Sea off the coasts of Mexico, Belize, Guatemala, and Honduras, making it a useful tool for transnational action planning in response to the *Sargassum* crisis. Using data from Sentinel-2 and Landsat-8/9 satellites, this platform offers high-resolution imagery coverage every five days, facilitating regular monitoring of the phenomenon and providing researchers and decision-makers with information for planning mitigation strategies. Another highlight of the platform is its focus on integrating multiple types of data. By incorporating detailed geospatial information, such as vector polygons depicting the extent of *Sargassum* rafts, and through the presentation of interactive tools that allow measurement of affected areas and dynamic modeling of the macroalgae trajectory, it provides a solid foundation for informed decision-making. These capabilities enable identification of the most affected areas and allow for more accurate planning of collection and mitigation efforts, a critical factor for an effective response to the massive arrival of *Sargassum*. On the other hand, the limitations of the platform include dependence on optical data, which can be affected by the cloudy conditions characteristic of the region, thus reducing the availability of usable images. However, the platform provides a solid framework upon which new technologies can be incorporated. These include radar sensors (SAR), which are not limited by adverse weather conditions, and thus allow monitoring with greater temporal resolution. In addition, the integration of citizen science, through data collected by users via mobile devices or drones, constitutes a relatively untapped opportunity that could serve to improve the efficiency of monitoring in specific coastal areas. Another highlight is the platform’s predictive capability based on dynamic models. Since these models evolve and are integrated with artificial intelligence and machine learning approaches, the accuracy of predicting *Sargassum* trajectory and behavior is expected to improve significantly. This will allow coastal authorities and communities to prepare earlier for the arrival of *Sargassum*, thereby potentially reducing its negative impacts. The LANOT platform represents a significant advance in *Sargassum* monitoring in the Mexican Caribbean. Its ability to integrate multiple data sources and provide up-to-date and detailed information, as well interactive decision-making tools, makes it a key tool for the management of this issue. Although it does face challenges such as dependence on optical data and the need to more actively integrate citizen science, these areas of opportunity offer a clear path for its future evolution. In a context where *Sargassum* continues to critically affect the region, the use of specialized platforms such as LANOT is essential not only to understand the phenomenon but also to efficiently mitigate its effects. This underscores the importance of continuing to develop and improve remote monitoring technologies applied to global-scale environmental problems, with a specific focus on regional needs, as is the case in the Mexican Caribbean.

## Data Availability

All data used in this study are provided by the platform and can be accessed from the following LANOT *Sargassum* project websites: the main project homepage (http://www.lanot.unam.mx/home/sargazo/), the map website (http://www.lanot.unam.mx/home/sargazo/visualizador/), and the file download website (http://www.lanot.unam.mx/home/sargazo/vistas/?monthyear=2022-07&region=s1).
